# Editorial: Emerging strategies in combatting and managing bacterial biofilms

**DOI:** 10.3389/fcimb.2023.1264346

**Published:** 2023-08-01

**Authors:** Reham Wasfi, Ashraf Zarkan, Samira M. Hamed

**Affiliations:** ^1^ Department of Microbiology and Immunology, Faculty of Pharmacy, October University for Modern Sciences and Arts (MSA), Giza, Egypt; ^2^ Department of Genetics, University of Cambridge, Cambridge, United Kingdom

**Keywords:** biofilm, microbiome, hospital-acquired infection (HAI), implanted medical devices, multidrug resistance (MDR), biofilm associated infections, catheters

Numerous microbes use biofilm formation as a mean of survival. Biofilms are multicellular communities in which microorganisms are encased in a protective matrix that enables them to endure challenging environments and resist traditional therapies. The widespread existence of biofilm-forming bacteria in various settings, including healthcare facilities, is made possible by their capacity to colonize a variety of biotic and abiotic surfaces. They pose a serious threat to human health because they can develop increasing resistance to traditional antibiotics and spread morbidity through both device- and non-device (tissue)-associated infections, as reviewed by Zhao et al. This microbial phenotype consequently became a significant concern in several fields, including public health and medicine.

Biofilms are involved in the pathogenicity of infectious diseases as well as the establishment of healthy microbiomes. Many bacterial species within the gut microbiome grow as biofilms, and disease outcome is greatly impacted by the location of the biofilms within the gastrointestinal tract (Miller et al., 2021). Hammouda et al. reported that hormonal drugs affect biofilm formation by selected gut microbiota such as *Bifidobacterium longum*, *Limosilactobacillus reuteri*, *Bacteroides fragilis*, and *Escherichia coli*, representing the four main phyla in the gut. Despite increasing the adhesion of *L. reuteri* to Caco-2/HT-29 cell line coculture, progesterone inhibited the biofilm development of the Gram-positive bacteria. In contrast, it increased the ability of Gram-negative bacteria to form biofilms and increased the adherence of *B. fragilis* to the cell lines coculture. Both estradiol and thyroxine displayed antibiofilm activity against *L. reuteri*. In the meantime, thyroxine boosted the capacity of *E. coli* to develop a biofilm.

The implication of biofilm-related multi-drug resistance (MDR) in hospital-acquired infections is a significant issue with increased rates of patient mortality and morbidity as well as economic burden, including high healthcare expenses and extended hospital stays (Assefa and Amare, 2022). Hu et al. reported the ability of the emerging opportunistic nosocomial pathogen *Elizabethkingia anophelis* to form biofilms. MDR phenotype was also exhibited by all isolates. The authors concluded that biofilm development and antibiotic resistance in *E. anophelis* are positively correlated. Such findings will provide the groundwork for future advancements in therapeutic approaches against *E. anophelis* infections. Due to its uncertain mechanism of antibiotic resistance and high mortality rate among nosocomial isolates, *E. anophelis* can be a serious concern to clinicians (Lin et al., 2019).

Another emerging opportunistic pathogen is *Brevundimonas* spp., which is reclassified from *Pseudomonas* spp. (Segers et al., 1994). Gricajeva et al. reported that the biofilm formed by this genus was responsive to treatment by antimicrobial inactivation using natural photosensitizers such as riboflavin (RF) and chlorophyllin (Chl). Importantly, this approach provides a new treatment strategy that does not drive resistance in treated microbial cells (Kashef and Hamblin, 2017).

Another nosocomial pathogen is *Acinetobacter bumannii* which is known for its high resistance and biofilm formation capacity (Abd El-Rahman et al., 2023; Hamed et al., 2023). *A. bumannii* was the subject of a study by Kong et al. who found that the dominance of some sequence types of *A. baumannii* is likely due to resistance to harsh conditions of oxidation, desiccation, and multiple antibiotics rather than their ability to form biofilm, while the non-dominant sequence types were characterized by high biofilm formation.

One approach for reducing the burden of biofilm-associated infections is the search for new and alternative therapies. Between 50-70% of nosocomial infections are caused by biofilm formation on implanted medical devices such as central venous catheters (CVCs) (Asker et al., 2021). Researchers have been looking for novel ways to develop biofilm-free implants via antibiofilm coating and impregnating devices with antibiofilm chemicals (Amer et al., 2022) as well as modifying the implant materials (Gayani et al., 2021). An et al. studied the impact of zinc dimethacrylate (ZDMA) modification of the polymethyl methacrylate (PMMA) denture base resin on its cytotoxic and antifungal activities as well as its surface and physicochemical properties. They confirmed that the ZDMA-modified PMMA showed higher thermal stability, surface hydrophilicity, and surface roughness without enhancing the adhesion of microbes. Additionally, it demonstrated strong antifungal action without causing any negative cellular consequences.

The influence of different surface modifications of implant materials based on cobalt–chromium–molybdenum (CoCrMo) on biofilms was studied by Paulitsch-Fuchs et al., where they compared three smooth surfaces (CoCrMo, CoCrMo polished, and CoCrMo TiN) and three rough surfaces (CoCrMo cpTi, CoCrMo porous coated, and CoCrMo TCP) to the unmodified base alloy. The authors found a relationship between surface roughness and biofilm structure, including proteins, polysaccharides, as wells as expression of biofilm-associated genes. Among all proposed surface modifications, the authors attributed the best performance in reducing biofilms to CoCrMo TiN and polished CoCrMo.

Amer et al. produced a potent biosurfactant from an endophytic *Bacillus amyloliquefaciens* that inhabited the Nile Papyrus. The biosurfactant showed promising antibacterial and antibiofilm activity against MDR global clones of *A. baumannii*. Up to 89.59% reduction in biofilm formation was achieved using sub-MICs of the extract. The potential of the biosurfactant to eradicate *A. baumannii* biofilms at concentrations equivalent to its MIC was also demonstrated by up to 87.3% biomass reduction. Three log10 reductions in the viable adherent bacterial count were achieved in a biosurfactant-impregnated CVC model. The authors linked this biosurfactant activity to several compounds explored by GC-MS analysis of the crude extract. The biosurfactant was hence, proposed as a potential strategy for reducing the burden of catheter-related blood stream infections (CRBSIs).

Early intervention is the key to reducing the clinical burden of biofilm-related infections, which can be facilitated by the early detection of biofilms. As biofilm detection is particularly challenging, innovative sensing, tracking, and diagnostic technologies are needed. The potential application of the BioFilm Ring Test (BRT)® in the diagnosis of biofilm-associated *Pseudomonas* respiratory infections was evaluated by Fernández-Barat et al. For this purpose, mucoid and nonmucoid *Pseudomonas aeruginosa* were recovered from the sputa of patients with bronchiectasis. The biofilm production index (BPI) of the isolates was determined using BRT at 5 and 24 hours. The authors concluded that the capacity of bacteria to form biofilms can be successfully determined using BRT in just five hours, and hence the test may be incorporated into clinical practise for the diagnosis of biofilm-related infections. Another application evaluated by the authors was the determination of the mucoid phenotype of *P. aeruginosa*. A BPI of less than 14.75 successfully predicted the mucoid phenotype at 5 h, but with low sensitivity and specificity (64% and 72%, respectively). A correlation between ciprofloxacin resistance and low BPI was also established by the authors, who recommended further investigation into the use of BRT to predict ciprofloxacin resistance.

To summarise, the contributions of these strategies in combatting and managing bacterial biofilms provide novel insights as well as potential therapeutic and preventive approaches that can be utilised in multiple clinical applications. The Research Topic is certainly of special interest to clinicians, dentists, and implant surgeons.

## Author contributions

RW: Writing – original draft. AZ: Writing – review & editing. SH: Writing – review & editing.
